# Oxygen effects on rhamnolipids production by *Pseudomonas aeruginosa*

**DOI:** 10.1186/s12934-018-0888-9

**Published:** 2018-03-09

**Authors:** Feng Zhao, Rongjiu Shi, Fang Ma, Siqin Han, Ying Zhang

**Affiliations:** 1 CAS Key Laboratory of Pollution Ecology and Environmental Engineering, Institute of Applied Ecology, Chinese Academy of Sciences (CAS), Shenyang, 110016 Liaoning China; 2 State Key Laboratory of Urban Water Resource and Environment, Harbin Institute of Technology, Harbin, 150090 China

**Keywords:** Mono-rhamnolipids, Di-rhamnolipids, HPLC–MS, *rhl*-genes, Emulsifying activity, *Pseudomonas aeruginosa*

## Abstract

**Background:**

Rhamnolipids are the most extensively studied biosurfactants and has been successfully used in various areas from bioremediation to industrial fields. Rhamnolipids structural composition decide their physicochemical properties. Different physicochemical properties influence their application potential. Rhamnolipids can be produced at both aerobic conditions and anaerobic conditions by *Pseudomonas aeruginosa*. This study aims to evaluate the oxygen effects on the rhamnolipids yield, structural composition, physicochemical properties and the *rhl*-genes expression in *P. aeruginosa* SG. Results will guide researchers to regulate microbial cells to synthesize rhamnolipids with different activity according to diverse application requirements.

**Results:**

Quantitative real-time PCR analysis revealed that *rhlAB* genes were down-regulated under anaerobic conditions. Therefore, strain *P. aeruginosa* SG anaerobically produced less rhamnolipids (0.68 g/L) than that (11.65 g/L) under aerobic conditions when grown in media containing glycerol and nitrate. HPLC–MS analysis showed that aerobically produced rhamnolipids mainly contained Rha-C_8_-C_10_, Rha–Rha-C_10_-C_12:1_ and Rha–Rha-C_8_-C_10_; anaerobically produced rhamnolipids mainly contained Rha-C_10_-C_12_ and Rha-C_10_-C_10_. Anaerobically produced rhamnolipids contained more mono-rhamnolipids (94.7%) than that (54.8%) in aerobically produced rhamnolipids. *rhlC* gene was also down-regulated under anaerobic conditions, catalyzing less mono-rhamnolipids to form di-rhamnolipids. Aerobically produced rhamnolipids decreased air–water surface tension (ST) from 72.2 to 27.9 mN/m with critical micelle concentration (CMC) of 60 mg/L; anaerobically produced rhamnolipids reduced ST to 33.1 mN/m with CMC of 80 mg/L. Anaerobically produced rhamnolipids emulsified crude oil with EI_24_ = 80.3%, and aerobically produced rhamnolipids emulsified crude oil with EI_24_ = 62.3%. Both two rhamnolipids products retained surface activity (ST < 35.0 mN/m) and emulsifying activity (EI_24_ > 60.0%) under temperatures (4–121 °C), pH values (4–10) and NaCl concentrations less than 90 g/L.

**Conclusions:**

Oxygen affected the *rhl*-genes expression in *P. aeruginosa*, thus altering the rhamnolipids yield, structural composition and physicochemical properties. Rhamnolipids produced at aerobic or anaerobic conditions was structurally distinct. Two rhamnolipids products had different application potential in diverse biotechnologies. Although both rhamnolipids products were thermo-stable and halo-tolerant, aerobically produced rhamnolipids possessed better surface activity, implying its well wetting activity and desorption property; anaerobically produced rhamnolipids exhibited better emulsifying activity, indicating its applicability for enhanced oil recovery and bioremediation of petroleum pollution.

## Background

Rhamnolipids are one of the most popular biosurfactants in current research [1, 2]. Due to their high surface activity and emulsifying activity, solubilization activity, low toxicity, and biodegradability [3, 4], rhamnolipids have been broadly applied in many different fields, such as microbial enhanced oil recovery (MEOR) [5–7], bioremediation of hydrocarbon pollutants and heavy metals [8–10].

Rhamnolipids are mainly produced by *Pseudomonas aeruginosa* [11, 12]. *P. aeruginosa* is one of facultative bacteria, which can grow and metabolize in both aerobic and anaerobic environments [13, 14]. Almost all studies about rhamnolipids production by *P. aeruginosa* were focused on aerobic conditions. However, some *P. aeruginosa* strains can also produce rhamnolipids under anaerobic conditions [15, 16]. What is the difference between aerobic production of rhamnolipids and anaerobic production of rhamnolipids? Information of oxygen effects (aerobic cultivation and anaerobic cultivation) on the rhamnolipids production is still scarce.

Rhamnolipids contain one or two rhamnoses attached to one or two β-hydroxyl fatty acids [11, 17]. Besides, the β-hydroxyl fatty acids chains vary significantly with lengths of 8, 10, 12, and 14 carbons [17]. In the rhamnolipids biosynthesis process, these groups may be randomly grouped to form different rhamnolipids homologues. Therefore, the structural composition of rhamnolipids are highly diverse [11].

The same *P. aeruginosa* strain can produce diverse rhamnolipids mixtures under different culture conditions. Déziel et al. reported strain *P. aeruginosa* 57RP produced different rhamnolipids mixtures grown on mannitol or naphthalene [18]. Nicolò et al. analyzed the different ratio of mono-rhamnolipids to di-rhamnolipids produced by *P. aeruginosa* L05 using four different carbon source [19]. Aerobic cultivation and anaerobic cultivation would also influence *P. aeruginosa* to produce rhamnolipids with different structural composition. Rhamnolipids structural composition decide their physicochemical properties. Changes in rhamnolipids composition can affect their properties, such as the CMC and emulsifying activity [20]. The different physicochemical properties of rhamnolipids will influence its application potential in different field. It is significant to understand the relationship between the rhamnolipids composition and its physicochemical activities, such as surface activity, emulsifying activity. The aim of this work was to investigate the effect of oxygen conditions (aerobic cultivation and anaerobic cultivation) on the rhamnolipids structural composition and physicochemical properties. Results will guide us to regulate microorganisms to synthesize highly active rhamnolipids according to different biotechnological application requirements.

In *P. aeruginosa*, the *rhlA* and *rhlB* genes arranged in an operon code the rhamnosyltransferase-1 (RhlAB) [21]. RhlA is involved in the synthesis of β-hydroxyl fatty acids. RhlB catalyzes dTDP-l-rhamnose and β-hydroxyl fatty acids to synthesis mono-rhamnolipids. The *rhlC* gene codes the rhamnosyltransferase-2 (RhlC) that catalyzes the second dTDP-l-rhamnose molecule adding to mono-rhamnolipids to form di-rhamnolipids [17]. In order to analyze the mechanism of oxygen effects on rhamnolipids production, expression of *rhlAB* genes and *rhlC* gene in *P. aeruginosa* under aerobic and anaerobic conditions was studied by quantitative real-time PCR (qRT-PCR).

In the present study, strain *P. aeruginosa* SG was used to produce rhamnolipids under aerobic or anaerobic conditions. This aims to evaluate the possible influence of oxygen on the rhamnolipids production in strain *P. aeruginosa* SG. Rhamnolipids production capacity of strain SG under aerobic conditions or anaerobic conditions was comparatively studied. The structure difference of two rhamnolipids products was analyzed by Fourier Transform infrared spectroscopy (FTIR) and high performance liquid chromatography–mass spectrometry (HPLC–MS). The different ratio of mono-rhamnolipids to di-rhamnolipids was also analyzed. The physicochemical properties of two rhamnolipids products were also comparatively evaluated, including surface activity, critical micelle concentration (CMC), emulsifying activity and stability. Application potential of two rhamnolipids products was also discussed.

## Methods

### Bacterial strain, medium and culture conditions

The bacterial strain *P. aeruginosa* SG (GenBank accession number KJ995745) isolated from oil reservoir environments was used for production of rhamnolipids under aerobic or anaerobic conditions, respectively [16]. The glycerol–nitrate (GN) medium with some improvements was used for culture of strain SG [22]. The GN medium contains 70.3 g/L of glycerol, 5.2 g/L of NaNO_3_, 6.9 g/L of K_2_HPO_4_·3H_2_O, 5.5 g/L of KH_2_PO_4_, 0.40 g/L of MgSO_4_∙7H_2_O, 1.0 g/L of NaCl, 1.0 g/L of KCl, 0.5 g/L of yeast extract and 0.13 g/L of CaCl_2_. The medium pH was adjusted to 6.8. All the chemicals and solvents (Sinopharm Chemical Reagent, Beijing, China) were analytical grade. The aerobic medium was dispensed into Erlenmeyer flasks and sterilized (121 °C, 20 min). The 500-mL Erlenmeyer flasks containing 250 mL GN medium were used for aerobic cultivation. Aerobic cultivation experiments were performed at 37 °C and 180 rpm for 6 days with three replicate. The anaerobic medium was prepared as follows. The medium was firstly boiled under a stream of oxygen-free N_2_ gas for 15 min. Then the medium was dispensed into serum bottles under a stream of oxygen-free N_2_ and sterilized (121 °C, 20 min). The 250-mL serum bottles containing 200 mL anaerobic GN medium were used for anaerobic cultivation. Anaerobic cultivation experiments were performed at 37 °C and 80 rpm for 10 days with three replicate.

### Comparative analysis of *rhlAB* and *rhlC* genes expression

Expression of the *rhlAB* genes and *rhlC* gene was investigated by quantification of mRNA transcripts using qRT-PCR. For total RNA extraction, 1 mL culture was sampled at the stationary growth phase. Because rhamnolipids yield reached maximum at the stationary growth phase. Strain SG was cultivated for 100 h at 37 °C, 180 rpm under aerobic conditions, and for 150 h at 37 °C, 80 rpm under anaerobic conditions, respectively. Cultivation experiments were performed with three replicate. Total RNA of samples was extracted using TaKaRa MiniBEST Universal RNA Extraction Kit (Takara, Japan) according to the manufacturer’s instructions. Random-primed reverse transcription was conducted using PrimeScript™ Double Strand cDNA Synthesis Kit (Takara, Japan) according to the manufacturer’s instructions.

The relative expression values of genes *rhlAB* and *rhlC* were obtained by the comparative CT (2^−ΔΔCT^) method [23–25]. 16S rRNA gene was used as endogenous control. The *rhlAB* genes, *rhlC* gene and 16S rRNA gene were amplified and quantified from cDNA using using a LightCycler instrument (Roche) and the SYBR Green I fluorophore protocol. The real-time quantitative PCR primers used for *rhlAB* genes, *rhlC* gene and 16S rRNA gene were as follows: rhlAB-F (TCAACGAGACCGTCGGCAAATACCT) and rhlAB-R (AATCCCGTACTTCTCGTGAGCGATG), rhlC-F (ATCCATCTCGACGGACTGAC) and rhlC-R (GTCCAGGTCGTCGATGAAC), 16S-341F (CCTACGGGAGGCAGCAG) and 16S-518R (ATTACCGCGGCTGCTGG). The 20 μL reaction volumes contained 10 μL of SYBR Premix Ex Taq™ II (Tli RNaseH Plus) (Takara, Japan), 0.8 μL of each primer (10 μM), 2 μL of template cDNA and 6.4 μL of PCR-grade water. Adding 2 μL of PCR-grade water instead of DNA template was used as a negative control. All amplifications were performed in triplicate. Thermal cycling consisted of one cycle of 95 °C for 3 min, followed by 40 cycles of 95 °C for 15 s, 55 °C for 30 s, and 72 °C for 45 s, followed by melt curve analysis to confirm the specificity of the amplicons. Fluorescence was measured during the last step of each PCR cycle. The relative expression values of *rhlAB* genes and *rhlC* gene were calculated using the comparative CT (2^−ΔΔCT^) method [23–25]. Relative expression values above and below 1 means a higher and a lower expression level under anaerobic conditions than that under aerobic conditions, respectively.

### Rhamnolipids extraction and quantification

The rhamnolipids products were extracted by the method referred to previous studies with some modifications [26, 27]. The bacterial cells were removed by centrifugation (10,000*g*, 10 min) to obtain supernatant. After heating at 80 °C for 30 min to denature the extracellular proteins, the pH value of supernatant was adjusted to 2.0 using 2 mol/L of HCl-water solution. Then the supernatant was kept at 4 °C for 12 h to precipitate rhamnolipids. Chloroform and methanol organic solvent (v/v, 2:1) was used for rhamnolipids extraction. The lower organic phase was collected and evaporated to dryness using a rotary evaporator (50 rpm, 45 °C). The thin yellowish rhamnolipids product was used for further analysis.

Oil spreading method was used for rhamnolipids quantification [28]. Briefly, 30 mL of distilled water was added into a 90 mm petri dish. Then 20 μL of crude oil was added to the surface of water to form a thin oil membrane. Then 10 μL of sample was gently dropped onto the center of the oil membrane. A clearly oil spreading circle was formed. The diameter of oil spreading circle was measured and recorded. All experiments were conducted three times. According to the diameter of formed oil spreading circle, the amount of rhamnolipids in culture was calculated from standard curves prepared with extracted rhamnolipids (100–800 mg/L). Therefore, samples which contain rhamnolipids high than 800 mg/L need to be diluted. The aerobically produced rhamnolipids extract and the anaerobically produced rhamnolipids extract were, respectively, used to prepare standard curves for quantify rhamnolipids in aerobic culture and anaerobic culture.

### Fourier Transform infrared spectroscopy analysis

The FTIR spectra of the aerobically produced rhamnolipids and the anaerobically produced rhamnolipids of strain SG were recorded by a NICOLET 380 FTIR spectrometer. Rhamnolipids product (10 mg) was mixed with 90 mg of KBr (spectral purity). The mixture was pressed with pressure of 25 Mpa for 25 s. The translucent pellet was used for FT-IR analysis with the resolution of 0.5 cm^−1^ and the wave number range from 400 to 4000 cm^−1^.

### HPLC–MS analysis

The aerobic produced rhamnolipids and the anaerobic produced rhamnolipids were dissolved into 10% acetonitrile (in water) solution containing 2 mM ammonium acetate, respectively. Two rhamnolipids solutions with concentrations of 500 mg/L were prepared for congener compositions analysis by high-performance liquid chromatograph-mass spectrometer (Waters, USA). The HPLC–MS analysis was performed according to Déziel’s method with minor modification [18]. A C18 reverse phase column (150 mm × 2 mm, particle size 5 μm) was used. An acetonitrile–water gradient from 10 to 60% was used as mobile phase. The HPLC flow rate was 0.6 mL/min. 10% of the flow was introduced into the mass spectrometer. Capillary voltage was set at 3.8 kV, cone voltage at 35 V and the source temperature was kept at 100 °C. The mass spectrometer was operated in the negative ion mode scanning 50–800 m/z range. Relative percentage content of each rhamnolipids homologue was calculated by area normalization method.

### Determination of critical micelle concentration

Rhamnolipids solutions (concentrations of 0–160 mg/L) were prepared to determine critical micelle concentration (CMC) values of aerobically produced rhamnolipids and the anaerobically produced rhamnolipids. The surface tension of each solution was measured. The relationship graphs between the surface tension and concentration of rhamnolipids were prepared. In the graph, the relevant rhamnolipids concentration at the turning point of surface tension is the CMC of rhamnolipids product, i.e., the surface tension reaches the lowest at the CMC value of rhamnolipids product.

### Analysis of surface activity and emulsifying activity

Two rhamnolipids-water solutions (concentrations of 1000 mg/L) were prepared using the aerobically produced rhamnolipids and the anaerobically produced rhamnolipids, respectively. Surface tension of rhamnolipids solutions was measured by a BZY-1 automatic surface tension meter (Shanghai equitable Instruments Factory, china). Emulsification index (EI_24_) was measured to evaluate emulsifying activity. Briefly, 4 mL of hydrophobic organics was mixed with 4 mL of rhamnolipids solutions in test tubes. The tubes were stirred at a vortex mixer for 2 min and placed at 37 °C for 24 h [16]. The EI_24_ (%) is the height of the emulsion layer (mm) divided by the total height of the mixture (mm) and multiplied by 100 [16]. The tested hydrophobic organics included kerosene, liquid paraffin, petroleum ether, isooctane, cyclohexane, *n*-hexane, benzene, olive oil and crude oil (Xinjiang Oilfield, China).

### Stability evaluation of two rhamnolipids product

Stability of the aerobically produced rhamnolipids and the anaerobically produced rhamnolipids were evaluated using 1000 mg/L of rhamnolipids solutions. 1 mol/L HCl and 1 mol/L NaOH were used to adjust pH values of samples. A series of 20 mL of two kinds of rhamnolipids solutions were treated under different temperatures (4, 25, 35, 45, 60, 80, 100 and 121 °C), different pH values (2, 4, 6, 8, 10 and 12) and different salinities (NaCl concentrations of 0, 3, 6, 9, 12, 15, 18, 21 and 25%). Surface tension and EI_24_ index (using crude oil as emulsion phase) were measured at 35 °C to evaluate the stability of the aerobically produced rhamnolipids and the anaerobically produced rhamnolipids.

## Results and discussion

### Rhamnolipids yield and *rhlAB* genes expression under two conditions

The diameters of the formed oil spreading circle are positively associated with the rhamnolipid concentrations (100–800 mg/L). For the aerobically produced rhamnolipid, the linear correlation model between the diameters of oil spreading circle (y) and rhamnolipid concentrations (x) was y = 0.0591x + 6.24, R^2^ = 0.9931. Another positive linear correlation model for the anaerobically produced rhamnolipid was y = 0.0616x + 5.9524, R^2^ = 0.9908. According to the diameter of formed oil spreading circle, the amount of rhamnolipids in culture was calculated using two positive linear correlation models. Strain SG obtained highest rhamnolipids yield under aerobic conditions at the 5th day. The rhamnolipids concentration in SG aerobic culture was 11.65 g/L. While the highest rhamnolipids yield of strain SG under anaerobic conditions was occurred at the 8th day. The rhamnolipids concentration in SG anaerobic culture was 0.68 g/L.

**Fig. 1:**
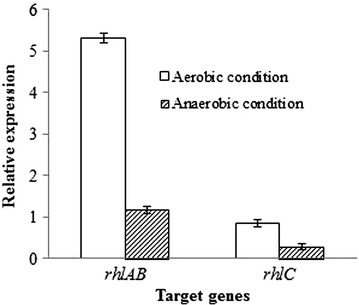
Expression of *rhlAB* genes and *rhlC* gene in *P. aeruginosa* SG under aerobic conditions or anaerobic conditions. The expression levels were determined by quantitative real-time PCR. 16S rRNA gene was used as endogenous control

The *rhlAB* gene codes the rhamnosyltransferase-1 catalyzing dTDP-l-rhamnose and β-hydroxyl fatty acids to synthesis mono-rhamnolipids [17]. As shown in Fig. 1, the relative expression value of *rhlAB* genes was lower (0.22-fold) under anaerobic conditions than under aerobic conditions. Previous studies on the transcriptome data of *P. aeruginosa* also showed that the expression of *rhlAB* genes in *P. aeruginosa* was down-regulated under anaerobic conditions compared with that under aerobic conditions [29, 30]. This may explain why strain SG produced less rhamnolipids under anaerobic conditions than aerobic conditions. In our previous study, increasing the copy numbers of *rhlAB* genes improved rhamnolipids yield under anaerobic conditions [31], indicating that up-regulating the anaerobic expression of *rhlAB* genes could enhance the anaerobic production of rhamnolipids.

### Fourier Transform infrared spectroscopy analysis of rhamnolipids

**Fig. 2:**
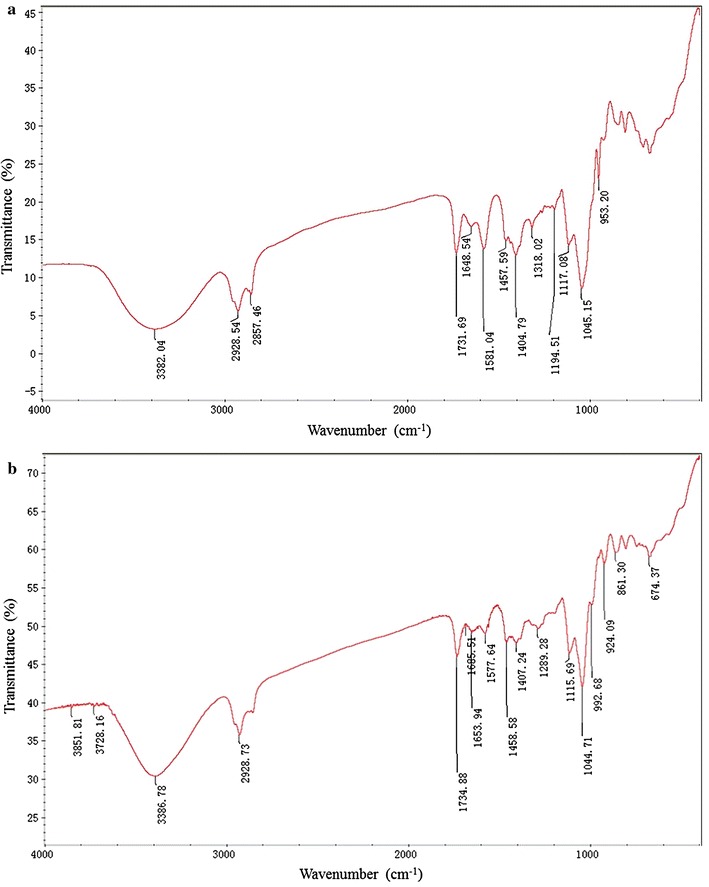
Fourier Transform infrared spectroscopy analysis of rhamnolipids products of strain SG: **a** rhamnolipids produced at aerobic conditions, **b** rhamnolipids produced at anaerobic conditions

The FT-IR spectra of two rhamnolipids products were shown in Fig. 2a, b, respectively. As shown in Fig. 2, the FT-IR spectra of two rhamnolipids products were very similar. Furthermore, both the two FT-IR spectra are similar to the reported spectra of rhamnolipids [26, 32, 33]. The characteristic absorption bands in two FT-IR spectra were explained as follows. The absorption bands around 2928, 2857, and 1457 cm^−1^ were, respectively, caused by the asymmetric stretching vibration of CH_2_, symmetric stretching vibrations of CH_2_, and scissoring vibrations of CH_2_ [33]. The absorption bands around 1731 cm^−1^ resulted from the ester groups. The fingerprint absorption area between 1458 and 1044 cm^−1^ are typical vibrations for carbohydrates. Results showed that two rhamnolipids products possessed the same structural group.

### Rhamnolipids compositions and *rhlC* gene expression analysis

**Fig. 3:**
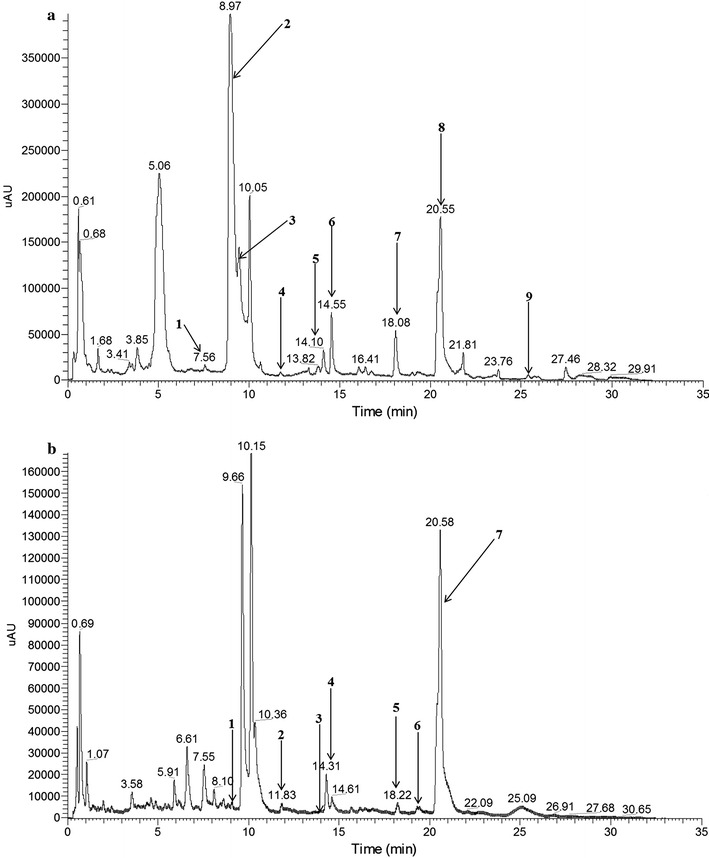
Liquid chromatogram results of rhamnolipids products of strain SG: **a** rhamnolipids produced at aerobic conditions, **b** rhamnolipids produced at anaerobic conditions

The liquid chromatogram results of two rhamnolipids produced under aerobic conditions and anaerobic conditions were shown in Fig. 3a, b, respectively. Because the analyzed samples were the crude extracts of rhamnolipids products, there were some impurity peaks in the chromatogram. The data analysis of HPLC–MS was according to Déziel’s study [18]. As shown in Fig. 3a, there were nine rhamnolipids homologues in the rhamnolipids compound produced under aerobic conditions. But the results of Fig. 3b showed that there were seven rhamnolipids homologues in the rhamnolipids compound produced under anaerobic conditions.

Both the two rhamnolipids products contained mono-rhamnolipids and di-rhamnolipids. As shown in Table 1, aerobically produced rhamnolipids contained three mono-rhamnolipids homologues and six di-rhamnolipids homologues. The main homologues in aerobically produced rhamnolipids are Rha–Rha-C_10_-C_12:1_, Rha–Rha-C_8_-C_10_, and Rha-C_8_-C_10_. As shown in Table 2, anaerobically produced rhamnolipids contained four mono-rhamnolipids homologues and three di-rhamnolipids homologues. The main homologues in anaerobically produced rhamnolipids are Rha-C_10_-C_12_ and Rha-C_10_-C_10_. Results confirmed that the structural compositions of two kinds of rhamnolipids products were different. According to previous studies, the concentration of rhamnolipid congeners is directly proportional to the relative abundance (%) obtained from HPLC–MS [18]. The ratio of mono-rhamnolipids to di-rhamnolipids (94.7%/4.3%) in anaerobically produced rhamnolipids was higher than that (54.8%/45.2%) in aerobically produced rhamnolipids.

**Table 1:** Structural composition of rhamnolipids produced by strain SG at aerobic conditions

Chromatographic peak number	Retention time (min)	Mass spectrum signal (m/z)	Rhamnolipids homologues	Relative abundance (%)
1	7.56	447	Rha-C_8_-C_8_	1.03
2	8.97	475	Rha-C_8_-C_10_	52.45
3	9.45	621	Rha–Rha-C_8_-C_10_	17.28
4	11.75	647	Rha–Rha-C_8_-C_12:1_	0.63
5	14.10	503	Rha-C_10_-C_10_	1.28
6	14.55	649	Rha–Rha-C_10_-C_10_	3.32
7	18.08	677	Rha–Rha-C_10_-C_12_	3.49
8	20.55	675	Rha–Rha-C_10_-C_12:1_	20.03
9	25.38	705	Rha–Rha-C_12_-C_12_	0.49

**Table 2:** Structural composition of rhamnolipids produced by strain SG at anaerobic conditions

Chromatographic peak number	Retention time (min)	Mass spectrum signal (m/z)	Rhamnolipids homologues	Relative abundance (%)
1	9.02	475	Rha-C_8_-C_10_	1.63
2	11.83	647	Rha–Rha-C_8_-C_12:1_	1.53
3	14.17	649	Rha–Rha-C_10_-C_10_	0.69
4	14.31	503	Rha-C_10_-C_10_	7.13
5	18.22	677	Rha–Rha-C_10_-C_12_	3.09
6	19.31	529	Rha-C_10_-C_12:1_	2.66
7	20.58	531	Rha-C_10_-C_12_	83.27

As shown in Fig. 1, the relative expression values of *rhlC* gene were lower (0.33-fold) under anaerobic conditions than under aerobic conditions. The *rhlC* gene, coding rhamnosyltransferase-2 that catalyzed mono-rhamnolipids to di-rhamnolipids, was down-regulated under anaerobic conditions, which may cause higher proportion of mono-rhamnolipids (94.7%) in anaerobically produced rhamnolipids than that (54.8%) in aerobically produced rhamnolipids.

### Critical micelle concentrations and surface activity

**Fig. 4:**
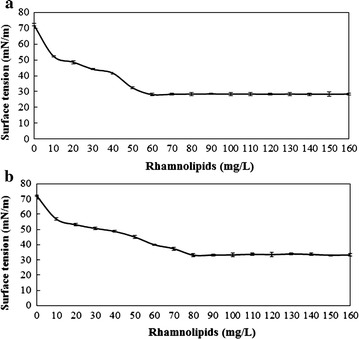
Relationship graphs between the surface tension and concentration of rhamnolipids products: **a** rhamnolipids produced at aerobic conditions, **b** rhamnolipids produced at anaerobic conditions

The aerobically produced rhamnolipids and the anaerobically produced rhamnolipids decreased the air–water surface tension from 72.3 to 27.9 mN/m and 33.1 mN/m, respectively. CMC is an important parameter to assess the interfacial activity of surfactants. Surface tension of surfactants reaches the lowest value at its CMC. As shown in Fig. 4, with the increase of rhamnolipids concentrations, the surface tension values rapidly decreased. Then the surface tension remained the minimum value with the continuous increase of rhamnolipids concentration. As shown in Fig. 4a, the surface tension of solutions of aerobically produced rhamnolipids reached the minimum value (28.3 mN/m) at the concentration of 60 mg/L, which revealed the CMC of aerobically produced rhamnolipids was 60 mg/L. In Fig. 4b, the surface tension of solutions of anaerobically produced rhamnolipids reached the minimum value (33.2 mN/m) at the concentration of 80 mg/L. The CMC of anaerobically produced rhamnolipids was 80 mg/L. While the chemical surfactants sodium dodecyl sulfate (SDS) has a CMC value of 2100 mg/L [27]. The CMC of rhamnolipids produced by other bacteria ranged from 10 to 230 mg/L [26, 27, 34]. The CMC represent the surface activity of surfactants. Both the CMC values of the aerobically produced rhamnolipids and the anaerobically produced rhamnolipids were lower than the CMC values of general chemical surfactants for 2 orders of magnitude, which showed better surface activity of biosurfactants. Surfactants with lower CMC values would reach the lowest surface tension with lower surfactant concentration, which also means the surfactant can change the surface property such as emulsification and foaming even at a relatively lower concentration [27].

### Emulsifying activity

**Fig. 5:**
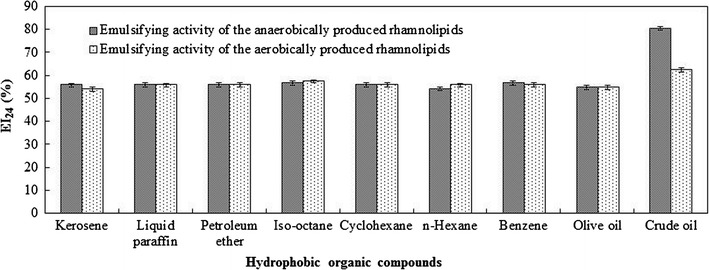
Emulsifying activity of the two types of rhamnolipids produced at aerobic or anaerobic conditions

Emulsifying activity of two rhamnolipids products were investigated using various hydrophobic organic compounds (including kerosene, liquid paraffin, petroleum ether, isooctane, cyclohexane, *n*-hexane, benzene, olive oil and crude oil). As shown in Fig. 5, both the two rhamnolipids products showed good emulsifying activity to all the tested hydrophobic organic compounds (all the EI_24_ > 56%). Anaerobically produced rhamnolipids has better emulsifying activity (EI_24_ = 80.3%) to crude oil than that (EI_24_ = 62.3%) of aerobically produced rhamnolipids. Using different hydrophobic organic compounds, rhamnolipids EI_24_ values were ranged from 53 to 90% [26, 27, 32]. Crude oil contains more complex components than other hydrophobic organic compounds, such as some components with molecular structure similar to rhamnolipids, which makes the rhamnolipids products show relative evident emulsifying activity for crude oil [35, 36]. The different structural composition makes the discrepancy in emulsifying activity for crude oil between aerobically and anaerobically produced rhamnolipids.

The good emulsifying activity of the rhamnolipids products makes them suitable for bioremediation of petroleum-contaminated sites and application in microbial enhanced oil recovery (MEOR). In petroleum-contaminated environments, the presence of rhamnolipids can increase the bioavailability of the petroleum hydrocarbons through emulsification [9, 37, 38]. Furthermore, rhamnolipids can be used as oil-displacing agent to mobilize the trapped oil in reservoirs based on its good oil emulsification activity [7, 39–41].

### Stability of two rhamnolipids products

Two rhamnolipids products were treated at different temperatures, pH and salinity. The surface tension and EI_24_ index of treated rhamnolipids solutions were measured at 35 °C to evaluate the stability of two rhamnolipids products. Two rhamnolipids products still reduced the surface tension of water to lower than 31 and 35.0 mN/m, respectively, after treated at temperatures (4–121 °C), pH values (2–10) and 0–150 g/L of NaCl. Two rhamnolipids products both had EI_24_ values (against crude oil) higher than 60% after treated at temperatures (4–121 °C), pH values (2–10) and 0–90 g/L of NaCl. There was no difference in the EI_24_ values among the different conditions. Results revealed that two rhamnolipids products were stable at different temperatures, pH and salinity. Although the structural compositions of two rhamnolipids products were different, both the two rhamnolipids products were thermo-stable and salt-tolerant. Both two rhamnolipids products are promising for biotechnology use in the complex and extreme environments, such as oil reservoirs, river sediment and petroleum contaminated soil.

### Application perspectives

Rhamnolipids consists of a hydrophilic moiety (one or two rhamnoses) and a hydrophobic moiety (saturated or unsaturated β-hydroxy fatty acids with different carbon chain lengths) [17]. Because of containing only one rhamnose, mono-rhamnolipids exhibits weaker hydrophilic ability and stronger lipophilic capability than di-rhamnolipids, i.e. mono-rhamnolipids has better emulsifying activity than di-rhamnolipids. Previous study reported that di-rhamnolipids was more effective than mono-rhamnolipids for desorption of triclosan from sediments [42], which confirmed that di-rhamnolipids had stronger surface activity than mono-rhamnolipids.

In this study, the ratio of mono-rhamnolipids to di-rhamnolipids (94.7%/4.3%) in anaerobically produced rhamnolipids was higher than that (54.8%/45.2%) in aerobically produced rhamnolipids. Therefore, the anaerobically produced rhamnolipids exhibited better emulsifying activity, but the aerobically produced rhamnolipids showed better surface activity, such as lower CMC value and lower surface tension. The unique properties of the anaerobically produced rhamnolipids validated strain *P. aeruginosa* SG as a promising biosurfactants producer for MEOR applications and environmental remediation of hydrocarbon pollutants. However, strain *P. aeruginosa* SG produced less rhamnolipids under anaerobic conditions than aerobic conditions. Therefore, future research would concentrate on how to enhance anaerobic production of rhamnolipids, such as using strain mutation, biosynthesis pathway regulation and gene manipulation. In future, we can regulate microorganisms to synthesize highly active rhamnolipids according to different biotechnology application requirements.

Rhamnolipids can improve the flow ability of trapped oil in the subsurface oil reservoirs and assist in the detachment of oil films from rocks based on its good oil emulsification activity [39, 40]. Furthermore, rhamnolipids can increase the bioavailability of the petroleum hydrocarbons through its emulsifying activity [9, 37, 38]. Anaerobically produced rhamnolipids of strain SG exhibited better emulsifying activity. Therefore, the anaerobically produced rhamnolipids would be more advantageous for applications in enhanced oil recovery and bioremediation of petroleum contaminated sites.

## Conclusions

Oxygen conditions affected the *rhl*-genes expression in *P. aeruginosa* SG, thus altering the rhamnolipids yield, structural composition and physicochemical properties. Rhamnolipids produced at aerobic or anaerobic conditions had distinct application potential in diverse biotechnologies. The *rhlAB* and *rhlC* genes were down-regulated under anaerobic conditions. Strain SG produced less rhamnolipids under anaerobic conditions than under aerobic conditions. Rhamnolipids produced at anaerobic conditions contained more mono-rhamnolipids. Aerobically produced rhamnolipids possesed better surface activity, so it is advantageous in the wetting and desorption applications. Anaerobically produced rhamnolipids exhibited better emulsifying activity, so it is promising for applied in MEOR and petroleum pollution bioremediation.
